# Case of disseminated histoplasmosis in a HIV-infected patient revealed by nasal involvement with maxillary osteolysis

**DOI:** 10.1186/s12879-017-2419-4

**Published:** 2017-05-05

**Authors:** A. C. Lehur, M. Zielinski, J. Pluvy, V. Grégoire, S. Diamantis, A. Bleibtreu, C. Rioux, A. Picard, D. Vallois

**Affiliations:** 1 Infectious and Tropical Diseases Department, University Hospital Bichat-Claude Bernard, APHP, 46 rue Henri Huchard, 75018 Paris, France; 2 Otorhinolaryngology Department, University Hospital Bichat-Claude Bernard, APHP, Paris, France; 3 Hematology Department, Meaux Hospital, Meaux, France; 4 General Medecine and Infectious Diseases Department, Melun Hospital, Melun, France

**Keywords:** Case report, Histoplasmosis, HIV, Maxillary osteolysis, Immunocompromized

## Abstract

**Background:**

Disseminated Histoplasmosis (DH) is a rare manifestation of Acquired Immune Deficiency Syndrome (AIDS) in European countries. Naso-maxillar osteolysis due to *Histoplasma capsulatum var. capsulatum* (*Hcc)* is unusual in endemic countries and has never been reported in European countries. Differential diagnoses such as malignant tumors, cocaine use, granulomatosis, vasculitis and infections are more frequently observed and could delay and/or bias the final diagnosis.

**Case presentation:**

We report the case of an immunocompromised patient infected by Human Immunodeficiency Virus (HIV) with naso-maxillar histoplasmosis in a non-endemic country. Our aim is to describe the clinical presentation, the diagnostic and therapeutic issues. A 53-year-old woman, originated from Haiti, was admitted in 2016 for nasal deformation with alteration of general condition evolving for at least 6 months. HIV infection was diagnosed in 2006 and classified at AIDS stage in 2008 due to cytomegalovirus infection associated with pulmonary histoplasmosis. At admission, CD4 cell count was 9/mm^3^. Surgical biopsies were performed and ruled out differential or associated diagnoses. Mycological cultures identified *Hcc* and Blood Polymerase Chain Reaction (PCR) for *Hcc* was positive. The patient was given daily Amphothericin B liposomal infusion during 1 month. *Hcc* PCR became negative in the blood under treatment, and then oral switch by itraconazole was introduced. Antiretroviral treatment was reintroduced after a 3-week histoplasmosis treatment. Normalization of naso-maxillar mucosa enabled a palatal prosthesis.

**Conclusion:**

Naso-maxillar histoplasmosis is extremely rare; this is the first case ever reported in a non-endemic country. Differential diagnoses must be ruled out by conducting microbiologic tools and histological examinations on surgical biopsies. Early antifungal treatment should be initiated in order to prevent DH severe outcomes.

## Background

Histoplasmosis is caused by a dimorphic saprophytic fungus, *Histoplasma capsulatum* which presents two variants: *Histoplasma capsulatum var. capsulatum* (*Hcc*) and *Histoplasma capsulatum var. duboisii* (*Hcd*)*. Hcc* is commonly found in soil contaminated with bird or bat droppings. Primary infection occurs through inhalation of spores [1]*.*

*Hcc* can take different clinical forms including Disseminated Histoplasmosis (DH) in immunocompromised individuals, such as patients with Acquired Immune Deficiency Syndrome (AIDS). DH is associated with AIDS in 70% to 90% of cases in endemic countries such as South American countries; however, it remains extremely rare in European countries. DH can involve various organs and naso-oral histoplasmosis is an uncommon manifestation of DH [2, 3].

This article reports the first case of nasal-oral histoplasmosis with naso-maxillar osteolysis in a patient infected by Human Immunodeficiency Virus (HIV) at AIDS stage, in a non-endemic country. Naso-maxillar osteolysis can be observed in tumors, infections, granulomatosis, vasculitis or drug exposure.

Our aim is to describe the clinical presentation, the diagnoses issues and the etiologic and functional treatments of naso-oral histoplasmosis.

## Case presentation

We report the case of a 53 year-old woman, originally from Haiti, living in France since 1989. She was hospitalized in the infectious diseases department of Bichat Claude Bernard University Hospital in Paris in March 2016 for alteration of general condition, fever and nasal deformity evolving for at least 6 months.

Her medical history is characterized by an HIV infection diagnosed in 2006, which was classified at AIDS stage in 2008 due to cytomegalovirus infection. At the same time, she suffered from pulmonary histoplasmosis that was treated with itraconazole. CD4 cell count nadir amounted to 7/mm^3^ in 2008. A first line of Highly Active Antiretroviral Therapy (HAART) was started 3 weeks after those diagnoses consisting in Efavirenz (EFV), Tenofovir Disoproxil Fumarate (TDF) and Emtricitabine (FTC). After 1,5 months the patient decided to interrupt this treatment, a second line of HAART was initiated 6 months later with nevirapine and TDF/FTC. She only took secondary prophylaxis with itraconazole for 6 months and then stopped being compliant with any treatment and was lost to follow-up over 7 years. No history of recent travel to endemic areas or contact with neither birds nor bats was reported. She never smoked, did not have excessive alcohol consumption and did not inhale any nasal drugs.

**Fig. 1:**
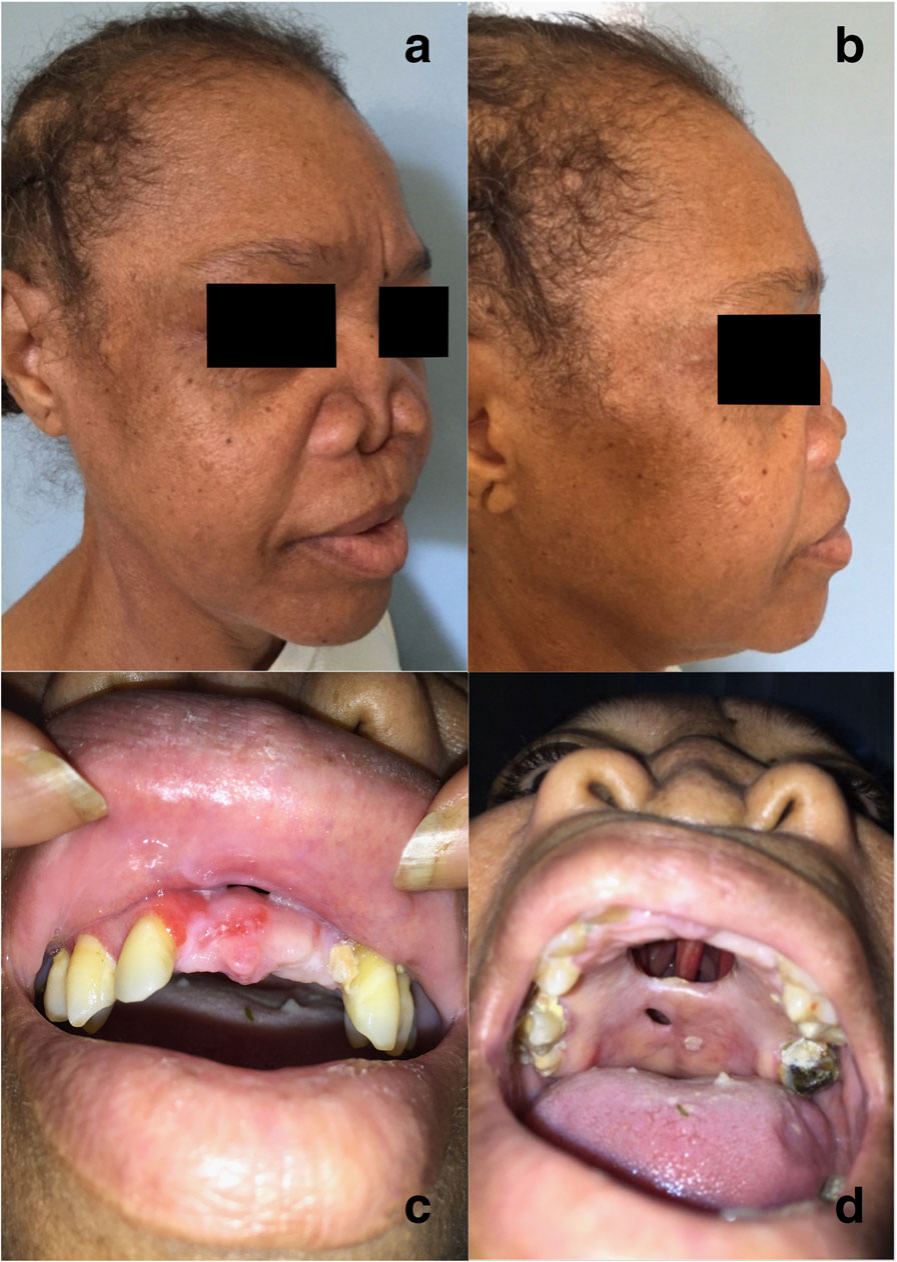
Clinical presentation of the case. **a**: Three-quarter picture of the face; **b**: Profile picture of the face; **c**: Face picture of the face showing lack of teeth 11, 21 and 22 which fall spontaneously; **d**: Bottom view of the face: nasal tip collapse, hard palate lysis and remaining nasal septum through the hole

**Fig. 2:**
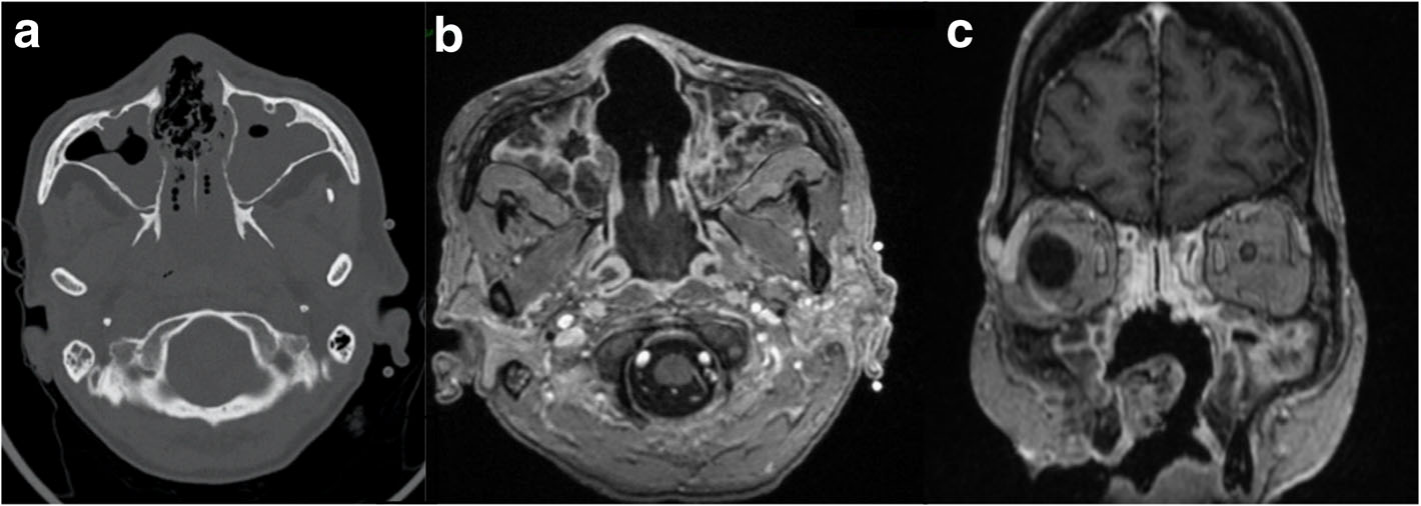
Imaging exploration. **a**: Sinus CT-scan showing bony nasal septum lysis and bilateral maxillary sinus opacities; **b** and **c**: Gadolinium-enhanced T1 weighted MRI (**b**: axial section; **c**: frontal section) showing bony nasal septum lysis and maxillar lysis without enhanced tumor mass

At hospital admission, physical examination revealed a nasal tip collapse, a disappearance of the nasal septum and a large perforation of the hard palate, resulting in naso-oral communication. Facial skin, alar and triangular cartilages were undamaged. However, maxillary teeth [4, 5] were loose, and teeth 11, 21 and 22 fell spontaneously. Mucosa of the nasal cavity was ulcerated with crusts and partially necrotic (Fig. 1). The sinus CT-scan and the magnetic resonance imaging performed showed: a complete loss of the nasal septum, a maxillar osteolysis, a communication between oral and nasal cavities and bilateral maxillary sinus opacities (Fig. 2). The chest X-ray showed an interstitial pneumonia. She did not manifest dyspnea, cough, cutaneous eruption, neurologic deficit, visceromegaly or lymphadenopathy. HIV viral load was 56,943 copies/ml and CD4 cell count was 9/mm^3^ (2%). In blood: Treponema Pallidum Hemagglutination Assay and Veneral Disease Research Laboratory, *Cryptococcus* antigen, *Toxoplasma* polymerase chain reaction (PCR), *Leishmania* PCR, galactomannan antigen, blood and mycological (×4) cultures were all negative. β-D-Glucane was positive at 338 pg/ml. Blood *Hcc* PCR was positive. In bronchoalveolar fluid: cultures, *Cryptococcus* antigen were negative, *Pneumocystis jirovecii* PCR level was barely positive (18,000 copies/mL), After 3 months, *Mycobacterium tuberculosis* sputum cultures were negative.

**Fig. 3:**
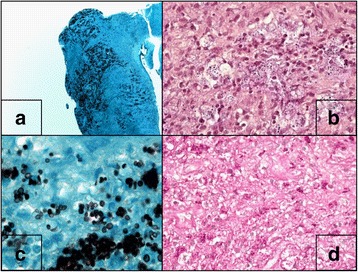
Histological analysis of naso-maxillar biopsy. **a** and **c**: Grocott staining; **b**: Gram Wegert staining; **d**: PAS staining

Surgical biopsies of oral, nasal and maxillary mucosa were performed and analyzed. Microscopic examination revealed fungal yeasts (Fig. 3), mycological cultures identified the presence of *Hcc* then confirmed by PCR.

Those tests enabled a final diagnosis of DH with naso-oral involvement in an AIDS-stage patient.

The patient received 200 mg per day of intravenous Amphotericin B (AmphoB) liposomal for 1 month. In the follow-up, we observed: negativation of blood histoplasmosis PCR in 10 days and clinical stabilization of naso-maxillary osteolysis with normalization of mucosa. Treatment was switched to 400 mg of oral itraconazole per day for a minimum of 1 year, in accordance with international guidelines [6].

HAART by ritonavir-boosted darunavir and TDF/FTC was reintroduced after 3 weeks of positive response to DH treatment. After 6 months, HIV viral load was undetectable and CD4 cell count at 40/mm^3^. At 9 months, biological results were stable and she had gained 17 k. Strategy of directly observed therapy permitted full compliance and the patient didn’t report any side effect with any treatment. Blood levels of itraconazole and HAART were both in the standards.

Ulceration and inflammation of the mucus decreased, enabling a palatal prosthesis to be implemented. A reconstructive surgery will be proposed to the patient upon completion of antifungal therapy and stabilization of HIV infection.

## Discussion

In immunocompromised individuals such as AIDS patients, especially when CD4 cell count is under 150/mm^3^, *Hcc* can produce a DH involving various organs, such as: liver, spleen, bone marrow, lymphnodes, gastrointestinal tract and central nervous system [2, 7]. Oral histoplasmosis is a rare lesion in DH and is hardly documented even in endemic regions. Palate, gingiva and tongue are the most frequently locations [3, 8]. Naso-maxillar histoplasmosis is extremely rare; we report the first case in a non-endemic country. It has previously been described that the lower CD4 T cell count is, the higher the probability of nasal and mucous lesion as manifestation of DH [7, 9, 10]. Our patient‘s very low CD4 T cell counts supports this hypothesis.

Other etiologies of naso-maxillar osteolysis have to be discussed and ruled out: i) non-infectious etiologies (malignant tumors, Wegener’s granulomatosis, sarcoidosis and consequences of drug exposure) and ii) other infectious etiologies (tuberculosis, leprosy, leishmaniosis, paracoccidioidomycosis and mucormycosis). The most frequent infectious cause of nasal lesion in AIDS patients is leishmaniosis.

The issue is that histoplasmosis can be confused with leishmaniosis for the following reasons: first of all *Histoplasma* yeasts could be interpreted as *Leishmania* amastigotes in direct examination; second, leishmaniosis frequently affects nasal mucosa [11, 12].

Because of its severity, mucormycosis must be explored and ruled out in this context, since very similar cases of maxillary osteolysis have been reported [13].

Chronic nasal ulcerations and nasal septum perforation can be symptoms of vasculitis, especially granulomatosis with polyangiitis (GPA) also known as Wegener’s granulomatosis. Unfortunately, the histological results show the classical triad of granulomatous inflammation, necrosis and vasculitis in up to 16% of cases of GPA. Diagnosis of GPA is based on a combination of clinical symptoms, histology and positive c-ANCA serology [14]. One case of naso-septal perforation related to Takayashu’s arteritis is reported [4]. In Behçet disease, patients with nasal mucosa involvement do not have more nasal manifestations than those without [15]. However no case of maxillary osteolysis caused by vasculitis has been reported in the literature so far.

An increasing cause of midface destructive lesions is cocaine exposure. Ischemia, mucociliar clearance modification, bacterial infection and lesions due to intranasal consumption lead to septal and hard palate perforation [16, 17]. In this context, a rise of c-ANCA may be possible, and may lead to a misdiagnosis with GPA. Nevertheless, necrosis and inflammation without vasculitis would be observed at histology examination [18].

At last, maxillary osteolysis can be observed in malignant tumors like mucoepidermoid carninoma, squamous-cell carcinoma and lymphoma [19, 20]. Extranodal NK/T-cell lymphoma associated with Epstein-Barr virus infection has to be highlighted because of its severity and the difficulties to diagnose it. It can lead to a misdiagnosis such as GPA [21, 22].

In literature only 18 cases of nasal mucosa involvement due to *Hcc* have been reported (Table 1). Three of them died because of delay in introducing treatment or non-adherence to treatment. Treatment was either AmphoB or itraconazole. Among patients, 12 had HIV related immunodeficiency, when it was reported, CD4 cell counts was always <150/mm^3^.

**Table 1:** Clinical characteristics of nasal and oral histoplasmosis in 18 patients

N°/Ref	Sex	Age (years)	Risk Factor	CD4 (/mm3)	Clinical Presentation	Diagnosis	Location	Treatment	Follow up (M: months)	Country
1 [31]	M	32	HIV infection	60	Pain	Histology	Nasal septum perforation	Amphotericin B then itraconazole	6 M, Improvement	Colombia
2 [31]	F	37	HIV infection	133	Nasal itch, epistaxis, nasal discharge	Histology	Nasal septum perforation	Itraconazole	2 M, Improvement	Colombia
3 [32]	M	59	HCV infection		Nose wound with crust	Not available	Nasal ulceration covered by crusts	Itraconazole	1 M, Improvement	Brazil
4 [33]	M	27	HIV infection	20	Fatigue, fever, skin lesions, dyspnea	Histology	Disseminated (nasal ulceration and cutaneous)	Amphotericin B then itraconazole	Improvement	Argentina
5 [34]	M	37	HIV infection	Not available	Fever, dysphagia, inflammatory and hemorrhagic nasal process	Histology	Destruction of nasal septum	No treatment	Death	Brazil
6 [35]	M	30	None		Nasal swelling	Histology	Nasal crusts	Itraconazole	Lost follow-up	India
7 [23]	F	79	Farmer		Fatigue, nasal obstruction	Histology	Nasal ulceration and crusts	Itraconazole	Lost follow-up	USA (Ohio)
8 [36]	M	37	Renal transplant, HBV/HCV coinfection		Nose septal destruction	Not available	Ulceration in mouth and nose	Amphotericin B then itraconazole	1 M, Improvement	Brazil
9 [37]	F	32	HIV infection	69	Fatigue, fever, nasal pain, nasal pyramid collapse	Histology	Destruction of the cartilaginous septum, columella and left nasal wing, and ulcerations	Amphotericin B	12 M, Improvement	Brazil
10 [38]	M	39	HIV infection	20	Nasal congestion, post nasal rhinorrhea, cough	Histology	Sinusitis, Skin rash, pulmonary involvement	Itraconazole	Improvement	USA (La)
11 [39]	F	49	HIV infection	Not available	Nasal obstruction	Histology and culture	Lesion on the nasal vestibule and septum	Not available	Death	Brazil
12 [39]	F	23	HIV infection	Not available	Not available	Histology	Crusts	Amphotericin B then itraconazole	Improvement	Brazil
13 [40]	M	43	HIV infection, farmer	15	Nasal regurgitation	Histology	Complete destruction of the nasal architecture, palatal perforation	Itraconazole	Improvement	Brazil
14 [41]	M	39	HIV infection	120	Nasal obstruction, post nasal rhinorrhea, headache, epistaxis	Histology	Papular and nodular rash, polypoid and inflammatory nasal mucosa in the right nasal fossa, bulging posterior wall of the nasopharynx	Amphotericin B	Improvement	Morocco
15 [42]	M	30	Adrenal insufficiency		Visual acuity impairment, nasal, septum and soft palate lesions	Histology	Endophtalmitis, nasal, septum and soft palate lesions	Amphotericin B (intraocular injection), itraconazole	12 W, Improvement	Argentina
16 [43]	NA	NA	HIV infection	Not available	Not available	Not available	Mouth, gingivae, hard palate, right maxillary sinus, right nasal cavity	Not available	Not available	South Africa
17 [44]	M	76	Chronic lymphocytic leukemia		Epistaxis and facial pain, facial erythema, swelling, and fever	Histology and culture	Ethmoïdal, sphenoidal and maxillary sinusitis	Amphotericin B then itraconazole	Improvement	Brazil
18 [45]	M	36	HIV infection, drug abuse	Not available	Fever, orodynia	Histology	Ulceration on the nasal bridge, necrotizing gingivitis and granulomatous oral lesion	Ketoconazole	4 M, Death	Brazil

Rizzi and *al*. reported a case of nasal histoplasmosis in an immunocompetent 79-year-old woman with nasal obstruction. Based on the histopathological examination, it was misdiagnosed with sarcoidosis and aggravation was observed on corticosteroids [23].

Generally speaking, diagnosis of histoplasmosis can be difficult because of its low frequency, its non-specific clinical presentation and some histological similarities with others causes of nasal damage [5, 11].

A review of literature reported only 72 patients with AIDS having presented histoplasmosis in Europe between 1984 and 2004 [24]. The diagnosis of histoplasmosis is based on mycological examination, antigen detection or detection of *Histoplasma* DNA by PCR. The limits are: the unlikelihood to see yeast with GIEMSA staining, the duration of the culture (up to 4 weeks) and its lack of sensitivity [25].

Antigen detection is quick (within 24 h) and allows monitoring response to treatment [26]. Unfortunately, this test is not available in non-endemic countries. Besides, false negatives results can occur especially in immunocompromised patients, as well as false positives results because of cross-reaction with other fungal infections [27]. In European countries, galactomannan antigen is usually used as a surrogate marker of histoplasmosis, especially on HIV patients. The sensitivity of this test is proportional to fungal load [28]. The negative result in our case study may be explained by the fact that it was conducted after treatment initiation.

Thanks to development of genetic diagnostic tools, histoplasmosis DNA can be detected by PCR either on blood sample or on tissue specimens. Its very good sensitivity, its 100% of specificity, and the rapidity to obtain the results make it the most reliable test as of today [29, 30]. Thus, if available, PCR must be performed on blood and tissue samples as soon as the diagnosis of histoplasmosis is suggested.

## Conclusion

Nasal histoplasmosis should be suspected for patients coming from endemic region, with deep immunosuppression (especially in AIDS patients).

Our patient is the first case of nasal architecture destruction by *Hcc* reported to date in a non-endemic region. After 1 year of treatment, she has had a really encouraging clinical course with good compliance illustrated by: controlled HIV infection, controlled DH and significant weight gain.

Given the very wide range of etiologies, the non-specific symptoms and the difficulty of histological analysis, a reliable diagnosis requires a thorough knowledge of maxillary osteolysis causes and an efficient cooperation and communication between surgeon, infectious disease clinician, mycologist and pathologist.

## Abbreviations

AIDS: Acquired immune deficiency syndrome
AmphoB: Amphotericin B
CMV: Cytomegalovirus
DRV: Darunavir
DH: Disseminated Histoplasmosis
EFV: Efavirenz
FTC: Emtricitabine
F: Female
GPA: Granulomatosis with Polyangiitis
HBV: Hepatitis B virus
HCV: Hepatitis C virus
HAART: Highly active antiretroviral therapy
*Hcc*: *Histoplasma capsulatum var. capsulatum*
*Hcd*: *Histoplasma capsulatum var. duboisii*
HIV: Human Immunodeficiency Virus
M: Male
M: Months
NVP: Nevirapine
PCR: Polymerase chain reaction
RTV: Ritonavir
TDF: Tenofovir disoproxil fumarate
VDRL: Veneral disease research laboratory
